# Comparative transcriptome analysis provides molecular insights into heterosis of waterlogging tolerance in *Chrysanthemum indicum*

**DOI:** 10.1186/s12870-024-04954-4

**Published:** 2024-04-10

**Authors:** Jiangshuo Su, Limin Zhao, Yingnan Yang, Yang Yang, Xuefeng Zhang, Zhiyong Guan, Weimin Fang, Fadi Chen, Fei Zhang

**Affiliations:** 1 State Key Laboratory of Crop Genetics & Germplasm Enhancement and Utilization, Key Laboratory of Biology of Ornamental Plants in East China, College of Horticulture, National Forestry and Grassland Administration, Nanjing Agricultural University, Weigang No.1, Nanjing, Jiangsu Province 210095 P.R. China; 2 Zhongshan Biological Breeding Laboratory, No.50 Zhongling Street, Nanjing, 210014 China

**Keywords:** Chrysanthemum, Heterosis, Waterlogging tolerance, RNA-seq, Overdominant expression

## Abstract

**Background:**

Heterosis breeding is one of the most important breeding methods for chrysanthemum. To date, the genetic mechanisms of heterosis for waterlogging tolerance in chrysanthemum are still unclear. This study aims to analyze the expression profiles and potential heterosis-related genes of two hybrid lines and their parents with extreme differences in waterlogging tolerance under control and waterlogging stress conditions by RNA-seq.

**Results:**

A population of 140 F_1_ progeny derived from *Chrysanthemum indicum* (Nanchang) (waterlogging-tolerant) and *Chrysanthemum indicum* (Nanjing) (waterlogging-sensitive) was used to characterize the extent of genetic variation in terms of seven waterlogging tolerance-related traits across two years. Lines 98 and 95, respectively displaying positive and negative overdominance heterosis for the waterlogging tolerance traits together with their parents under control and waterlogging stress conditions, were used for RNA-seq. In consequence, the maximal number of differentially expressed genes (DEGs) occurred in line 98. Gene ontology (GO) enrichment analysis revealed multiple stress-related biological processes for the common up-regulated genes. Line 98 had a significant increase in non-additive genes under waterlogging stress, with transgressive up-regulation and paternal-expression dominant patterns being the major gene expression profiles. Further, GO analysis identified 55 and 95 transgressive up-regulation genes that overlapped with the up-regulated genes shared by two parents in terms of responses to stress and stimulus, respectively. 6,640 genes in total displaying maternal-expression dominance patterns were observed in line 95. In addition, 16 key candidate genes, including *SAP12*, *DOX1*, and *ERF017* which might be of significant importance for the formation of waterlogging tolerance heterosis in line 98, were highlighted.

**Conclusion:**

The current study provides a comprehensive overview of the root transcriptomes among F_1_ hybrids and their parents under waterlogging stress. These findings lay the foundation for further studies on molecular mechanisms underlying chrysanthemum heterosis on waterlogging tolerance.

**Supplementary Information:**

The online version contains supplementary material available at 10.1186/s12870-024-04954-4.

Heterosis refers to the phenomenon where hybrids exhibit superior traits compared to their parents in agronomic characteristics [1]. Heterosis breeding has been proposed and applied for over a century, but there is still no complete and clear explanation for the formation mechanism of heterosis [2]. The overdominance theory proposes that heterosis arises from favorable interactions between alleles in heterozygotes, leading to heterozygotes performing better than homozygotes [3, 4]. Overdominant effects have been observed in various heterosis traits in different plants, such as rice [5], maize [6], and tomato [7]. The dominance theory suggests that heterosis comes from the mask of unfavorable recessive genes by favorable dominant genes at heterozygous loci [8, 9]. This theory has found evidence in many hybrid species of diploid plants [10, 11]. The epistasis theory suggests that the interaction between multiple alleles controlling major traits gives rise to multiplicative effects, and these genes controlling sub-traits collectively contribute to heterosis [12]. Evidence of epistasis involvement in heterosis has been found in rice [13, 14] and maize [15, 16]. The dosage balance in gene regulation hypothesis proposes that the dosage effects of non-additive genes modulate heterosis by affecting dosage-dependent regulatory systems, with transcription factors, chromatin proteins, and signaling transduction cascade members being the major factors of dosage-dependent regulatory systems [17–19]. Recent studies suggest that it is challenging to identify common genomic regions that explain heterosis in different hybrid combinations. Therefore, focusing on the analysis of gene expression patterns may provide a better understanding of heterosis[20]. These mechanisms described by different theories can not be fully applied to every heterosis trait. The formation mechanisms of heterosis for some traits may also involve multiple effects [2, 21], as the phenomenon of heterosis is based on the non-linear effects of multiple heterozygous gene combinations of parental genetic differences [12, 22], which also restrict individual exploration of heterosis from a genetic perspective.

Due to potential interactions within the parental genomes, the gene expression patterns and levels of hybrids are often taken as a focus in dissecting heterosis. For example, it was found that the yield heterosis might be associated with genes related to energy metabolism and sugar metabolism pathways among differentially expressed genes (DEGs), according to RNA-seq performed on rice hybrids at the panicle primordium differentiation stage and grain filling stage [23]. In another transcriptome analysis of the hybrid rice Xieyou 9308, carbohydrate metabolism and hormone signaling pathway-related genes were identified to potentially regulate the root growth heterosis during the tillering and heading stages [24]. The approach of unraveling heterosis based on DEGs analysis of transcriptome has also been applied to other plants such as soybean [25], upland cotton [26], and pepper [27]. Non-additive genes, which exhibit significantly different expression levels compared to the median parental value of hybrids, may be the key to understanding the formation of heterosis [28]. A recent study on Easter lily (*Lilium longiflorum*) found that most DEGs showed overdominant expression, and 28 of these genes may be involved in regulating the plant height and leaf elongation heterosis [29]. In contrast, a study on cabbage head heterosis found that over 81% of the DEGs exhibited dominant expression [30]. Transcriptome analysis revealed that 63.1% of DEGs in the super-hybrid rice WFYT025 exhibited overdominant expression, suggesting that these overdominant genes might be the major contributors to the grain yield heterosis of WFYT025 [31]. Compared to intensive focuses on growth and yield advantages, there has been less research on heterosis for plant stress tolerance. A study on the heat tolerance heterosis of maize hybrid An’nong 591 found that 5400 non-additive genes were specifically expressed in the hybrid under heat stress. Among them, 33 DEGs enriched in heat response terms were identified, demonstrating the important role of non-additive expression patterns in the heat tolerance heterosis of maize [32].

Waterlogging is one of the most important abiotic stresses affecting plant growth and development [33]. Chrysanthemum (*Chrysanthemum morifolium* Ramat.) is a perennial herbaceous flower with shallow roots and poor waterlogging tolerance. Even short periods of waterlogging can cause wilting, yellowing of leaves, browning, and rotting of roots, hence decreasing its ornamental quality and yield. Prolonged waterlogging can even lead to extensive mortality in cultivated chrysanthemum [34]. Due to the polyploidy and high heterozygosity of cultivated chrysanthemums, the application of molecular breeding technologies for chrysanthemums is still hindered. Accordingly, hybrid breeding is generally regarded as an effective way to improve the waterlogging tolerance of chrysanthemums [35]. Some progress has been made on waterlogging tolerance in chrysanthemums. Using the membership function method, researchers established a comprehensive evaluation system for waterlogging tolerance in chrysanthemums [36]. In analyzing the interrelationships among combining ability, genetic distance, and heterosis using a 4 × 3 incomplete diallel cross, it was found that six growth and biomass-related traits were mainly controlled by non-additive gene effects [37]. By constructing a genetic linkage map using 162 F_1_ progeny, 37 unconditional QTLs, 51 conditional QTLs, and several epistasis QTLs associated with waterlogging tolerance were successfully identified [38]. Several wild *Chrysanthemum* species give access to resistance breeding by providing pivotal candidate resistance genes that are not usually absent in cultivated chrysanthemum [34, 39]. Wang et al. (2013) obtained hybrid offspring with stronger waterlogging tolerance through interspecific hybridization between *C. morifolium* cv. ‘Nannong Yinshan’ and *C. zawadskii*, demonstrating that interspecific hybridization is an effective way to improve the waterlogging tolerance of cultivated chrysanthemums [40]. To date, little is known about the mechanisms of heterosis on waterlogging tolerance, and the causal genes responsible for waterlogging tolerance urgently need to be dissected in chrysanthemums. *Chrysanthemum indicum* has been regarded as making significant contributions to the origin of cultivated chrysanthemum, which has wide geographical distribution, multiple ecotypes, and high genetic diversity, severing a model species for hexaploidy chrysanthemum [41].

In this study, we assessed the genetic variation and heterosis of waterlogging tolerance in an F_1_ population derived from waterlogging-tolerant *C. indicum* (Nanchang) and waterlogging-sensitive *C. indicum* (Nanjing) [42]. To further explore the formation mechanism of waterlogging tolerance heterosis in chrysanthemums, two hybrid offspring, lines 98 and 95, exhibiting extreme differences in waterlogging tolerance, as well as two parents, were selected to investigate the global transcriptomes before and after waterlogging stress using RNA-seq. Differentially expressed transcripts and their expression patterns were analyzed, and 16 key candidate genes assigned to GO terms for response to stress and hormone response were identified. Our findings provide new insights into the mechanisms underlying waterlogging tolerance heterosis in chrysanthemums and contribute to the utilization of heterosis in future breeding programs.

## Materials and methods

### Materials

The experimental materials consisted of the highly waterlogging-tolerant maternal parent, *C. indicum* (Nanchang) (termed NC), and the waterlogging-sensitive paternal parent, *C. indicum* (Nanjing) (termed NJ), along with 140 F_1_ lines. Hybridization was conducted from October to November 2018, with the maternal plants bagged immediately after emasculation and artificial pollination. Mature and fully developed seeds were harvested two months after pollination and stored at room temperature. In March 2019, the seeds were sown in polystyrene foam trays containing a substrate mixture of vermiculite and perlite (3:1 ratio). After germination, which took approximately 60 days, the seedlings were transplanted into the field and sequentially numbered using Arabic numerals. Two rounds of top pruning were performed on the seedlings to generate sufficient cuttings for waterlogging tolerance identification. All the materials were preserved at the Chrysanthemum Germplasm Resource Preservation Center of Nanjing Agricultural University (118.985°N, 32.068°E).

### Waterlogging treatment and phenotypic evaluation

The potted waterlogging experiments were conducted in a greenhouse from August to October 2019 and June to August 2020. Healthy, uniform plants of two parents and 140 F_1_ progeny were selected for the experiments. The 6 cm cuttings were planted in 32-hole high-footed plug trays filled with a sterile substrate mixture of perlite and vermiculite in a ratio of 1:3. Once the cuttings developed roots and reached approximately ten leaves, they were transferred to turnover boxes (65 cm × 43 cm × 16 cm) for further treatment. The experiments consisted of control and treatment groups. For both the parents and their offspring, the treatment groups were replicated 10 plants per individual, while the control groups were replicated 6 plants per individual. The waterlogging stress was imposed by maintaining 3 cm above the soil surface, and water was supplied regularly to maintain the stress level [36, 43]. The control plants were subjected to normal watering management. The temperature during the two experimental periods was controlled at 20–25°C, with a relative humidity of 80% and a photoperiod of 16 h.

After 9 days of waterlogging treatment, scores were recorded based on four morphological indicators: leaf color (1–7 points), leaf shape (1–5 points), stem color (1–4 points), and stem shape (1–4 points) to assess the appearance of the plants following waterlogging. Since the control group exhibited good growth, the Score was only performed on the treatment group. The score criteria were chosen according to the method described in a previous study [36], where the score values were derived from a combination of four morphological indicators. Six growth parameters, i.e., shoot height (SH), root length (RL), shoot fresh weight (SFW), shoot dry weight (SDW), root fresh weight (RFW), and root dry weight (RDW), were measured for both the control and treatment groups. The measurements of these growth parameters were taken immediately after the ending of stress treatment. SH and RL were measured using a ruler, while SFW and RFW were measured using an electronic balance. Then, the samples were placed in envelopes and dried at 105°C until a constant weight was reached. SDW and RDW were measured using an electronic balance. The average of ten replicates were used as the phenotypic values of each individual for the Score and 6 biomass traits.

To describe the differences in waterlogging tolerance among the parents and 140 F_1_ lines, the waterlogging tolerance index (WI = treatment group value/control group value) was calculated for each measured trait. Furthermore, the waterlogging tolerance of each entry in *C. indicum* F_1_ population was evaluated using the membership function method via the following formula: *X*_*i*_ = (*X* − *X*_*min*_) / (*X*_*max*_ − *X*_*min*_) × 100%, where *X*_*i*_ represents the membership function value of the *i*-th traits, *X* represents the waterlogging tolerance index value of the *i*-th traits, and *X*_*max*_ and *X*_*min*_ represent the maximum and minimum values of the waterlogging tolerance traits for the i-th traits, respectively. The average membership function values of waterlogging tolerance traits (MFVW) were used to represent the waterlogging tolerance of each entry, where a higher MFVW indicates more tolerance. Following the approach by Yan [44], the waterlogging tolerance grade of the F_1_ population was classified into five categories based on the MFVW and standard deviation (*SD*): highly waterlogging-tolerant (HWT, *X*_*i*_ ≥ MFVW + 1.64*SD*), waterlogging-tolerant (WT, MFVW + 1*SD* ≤ *X*_*i*_ ˂ MFVW + 1.64*SD*), moderately waterlogging-tolerant (MWT, MFVW − 1*SD* ≤ *X*_*i*_ ˂ MFVW + 1*SD*), waterlogging-sensitive (WS, MFVW − 1.64*SD* ≤ *X*_*i*_ ˂ MFVW − 1*SD*), and highly waterlogging-sensitive (HWS, *X*_*i*_ ˂ MFVW − 1.64*SD*), where *X*_*i*_ represents the membership function value of the *i*-th hybrid line.

### Phenotypic data processing and analysis

The calculation of heterosis was based on the method described by Zhang et al. [45], using two parameters: mid-parent heterosis (MPH) and high-parent heterosis (HPH). Data input and organization were performed using Microsoft Excel 2019 software. Descriptive statistical analysis, correlation analysis, and significance analysis of heterosis were conducted using SPSS 24 software.

### Sample preparation and RNA sequencing

Root samples were collected from two parental plants, *C. indicum* (Nanjing) and *C. indicum* (Nanchang), as well as two F_1_ lines with extreme differences in waterlogging tolerance, lines 98 (HWT) and 95 (HWS), after 0 h and 12 h waterlogging treatment. Three biological replicates were prepared, with each replicate consisting of three consistent plants. Approximately 0.5 g of each sample was collected after mixed-grinding, rapidly frozen in liquid nitrogen, and then stored at -80°C. Total RNA was extracted using the Trizol reagent kit (Invitrogen, Carlsbad, CA, USA) according to the manufacturer’s instructions. The RNA quality was evaluated using the Agilent 2100 Bioanalyzer (Agilent Technologies, Palo Alto, CA, USA), and RNase-free agarose gel electrophoresis was performed to confirm the integrity of the RNA. After total RNA extraction, the rRNA was removed using the Ribo ZeroTM Magnetic Kit (Epicentre, Madison, WI, USA) to enrich for mRNA, which was then reverse-transcribed into cDNA using random primers. The cDNA fragments were purified using the QiaQuick PCR Purification Kit (Qiagen, Venlo, the Netherlands), followed by end repair, the addition of A bases, and ligation to Illumina sequencing adapters. Gel electrophoresis was performed to select the appropriate size range of the ligated products, followed by PCR amplification. Sequencing was conducted using the Illumina NovaSeq 6000 platform (Gene Denovo Biotechnology Co., Guangzhou, China).

### Sequence assembly and data analysis

The raw reads obtained from sequencing were subjected to quality control using the fastp software [46]. This process involved the removal of reads containing adapters and reads consisting entirely of A bases. Additionally, low-quality data (more than 50% base quality value Q ≤ 20) was filtered. This resulted in the generation of clean reads. The Trinity software was used for read assembly [47]. Trinity first merged reads with a certain length overlap to generate longer fragments. These assembled fragments, obtained through read overlap relationships and devoid of N bases, were considered assembled unigenes. The quality of the assembly results was evaluated using metrics such as the N50 value, sequence length, and Benchmarking Universal Single-Copy Orthologs (BUSCO) analysis (http://busco.ezlab.org/). To annotate the unigene sequences, a blastx search was performed against protein databases including NR, SwissProt, KEGG, and COG/KOG, with an E-value cutoff of < 1E-5. This allowed the identification of the protein with the highest sequence similarity to the given unigene, providing protein functional annotation information for the unigene. For gene differential expression analysis, the input data consisted of read counts obtained from gene expression level analysis. The edgeR software was utilized for differential analysis between samples [48], while the DESeq2 software was employed for differential analysis between groups [49]. Genes with a false discovery rate (FDR) ≤ 0.05 and a fold change ≥ 2 were selected as significant DEGs. The assembled unigenes were quantified using the RSEM software [50], and gene expression levels were calculated using the reads per kilobase of transcript per million mapped reads (RPKM) method. Transcriptome sequencing and analysis were conducted by Guangzhou Jidio Biotechnology Co., Ltd. Venn diagrams and heatmaps of gene expression levels were generated using the omicsmart platform tools.

### Quantitative real-time PCR validation of RNA sequencing

Six DEGs were randomly selected to validate the reliability of RNA sequencing data. Primers for gene validation were designed using Primer 5.0 software. The reference gene, the *EF1α* gene of *C. indicum*, was selected for normalization. The qRT-PCR procedure followed the methods described by Ren et al. [51]. Each sample was subjected to three technical replicates. The primer sequences used are listed in Table S1.

### GO and KEGG pathway enrichment analysis

The DEGs were mapped to various terms in the Gene Ontology (GO) database (http://www.geneontology.org/), and the number of genes associated with each term was calculated. This allowed for the identification of gene lists and the statistical determination of the number of genes associated with specific GO functions. GO terms that met the threshold of Q value ≤ 0.05 were defined as significantly enriched terms. Pathway analysis was performed using the Kyoto Encyclopedia of Genes and Genomes (KEGG) web server (http://www.kegg.jp/) [52, 53]. After multiple testing corrections, pathways with a Q value ≤ 0.05 were defined as significantly enriched pathways.

### Classification and identification of gene expression patterns

The average expression values of three biological replicates of two hybrids were compared with the mid-parent values after 0 h and 12 h of waterlogging stress, respectively. Short Time-series Expression Miner software was used to analyze the gene expression patterns [54]. The classification of gene expression patterns in hybrids was based on the study by Swanson-Wagner et al. [55]. Genes were categorized into eight significant types (Q-value ≤ 0.05) based on the difference in expression levels between hybrids and parents, i.e., additive (hybrid gene expression equal to the average of parents), two types of overdominance (hybrid gene expression significantly higher or lower than parents), two types of high-parent dominance, and two types of low-parent dominance (hybrid gene expression equal to one parent and significantly higher or lower than the other parent).

## Results

### Waterlogging tolerance identification of *C. indicum* F_1_ progeny

**Fig. 1:**
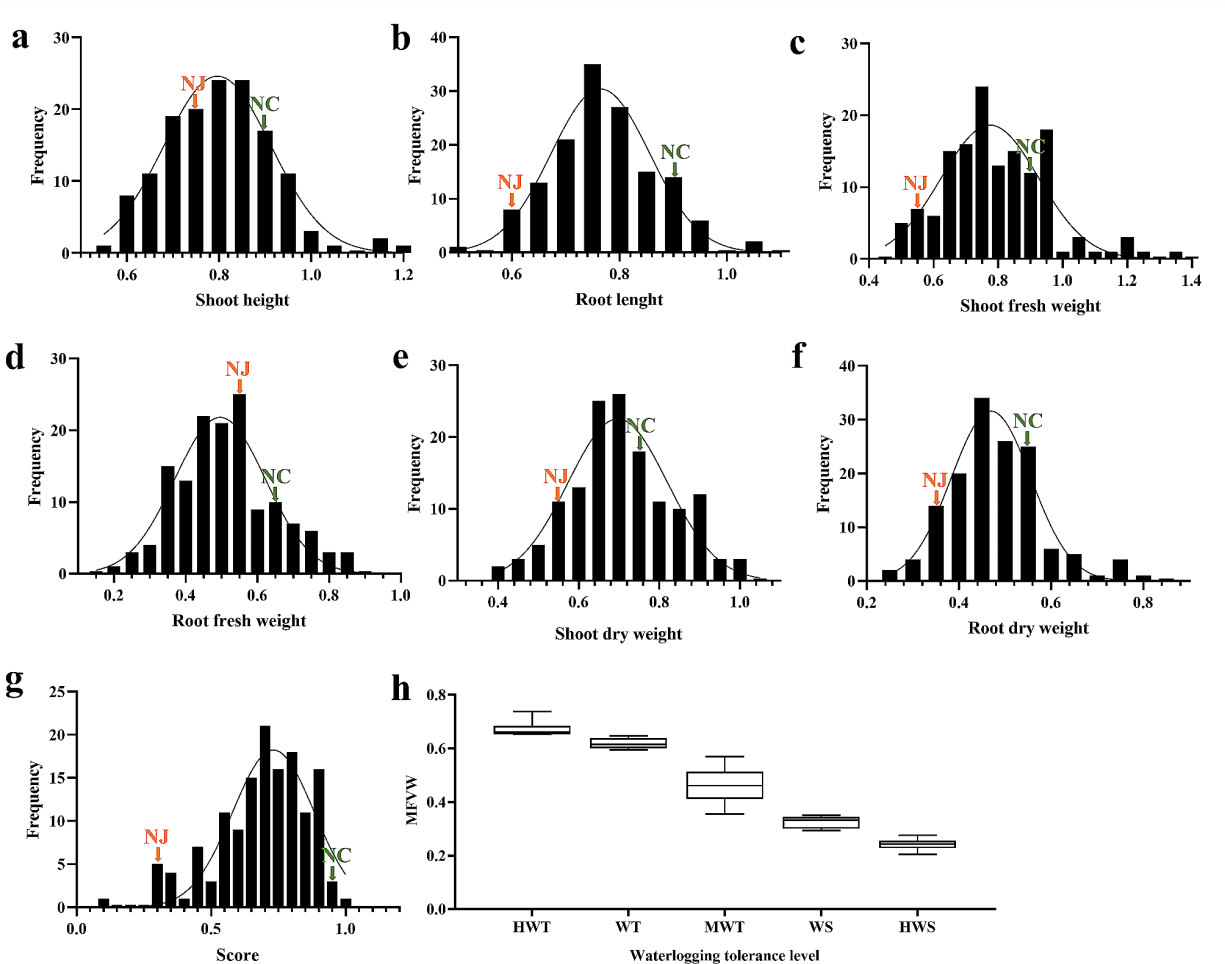
Frequency distribution of seven waterlogging tolerance traits and box plot of MFVW in the F_1_ population derived from *C. indicum* (Nanchang) and *C. indicum* (Nanjing). **a** Shoot height. **b** Root length. **c** Shoot fresh weight. **d** Root fresh weight. **e** Shoot dry weight. **f** Root dry weight. **g** Score. The green and orange arrowheads in panel a-g indicate the two parents: NC, *C. indicum* (Nanchang); NJ, *C. indicum* (Nanjing). **h** Box plot showing the variation of MFVWs among the five waterlogging tolerance levels in F_1_ lines

Statistical analysis of the F_1_ population after 9 days of waterlogging stress revealed a wide range of phenotypic variations among the seven investigated traits (Table S2, Fig. 1a-g). The coefficients of variation ranged from 14.47 to 59.38%, with SFW and SDW showing higher variability than other traits, indicating a more pronounced response of the root system to waterlogging stress. Based on their MFVWs, 68% of the F_1_ progeny were classified as moderately waterlogging-tolerant, while 7 and 6 individuals belonged to the HWT and HWS categories, accounting for 5.63% and 4.93%, respectively (Table S3, Fig. 1h). The phenotypes of five representative F_1_ lines after 9 days of treatment are shown in Fig. S1. Heterosis analysis revealed significant positive mid-parent heterosis in the F_1_ population for score, RL, SFW, SDW, and RDW, ranging from 10.09 to 34.41%. MFVW has the weakest positive mid-parent heterosis (2.93%) and is insignificant. The high-parent heterosis for all waterlogging tolerance traits in this F_1_ population was negative (Table 1). However, several individuals with positive and negative transgressive segregation were found for these traits (Fig. 1), indicating a significant transgressive segregation phenomenon in the F_1_ generation.

**Table 1:** Heterosis and MFWV of waterlogging tolerance traits in *C. indicum* (Nanchang), *C. indicum* (Nanjing), and their F_1_ hybrids

Traits	*C.indicum* (Nanchang)	*C.indicum* (Nanjing)	MPV	F_1_ population			
				*CV*	Mean	MPH/%	HPH/%
Score	0.95	0.3	0.63	24.33%	0.69	10.08**	-27.58**
SH	0.59	0.34	0.46	47.40%	0.38	-17.03**	-34.54**
RL	0.71	0.12	0.41	37.67%	0.46	11.15**	-35.01**
SFW	0.44	0.08	0.26	51.77%	0.35	34.41**	-21.12**
RFW	0.8	0.54	0.67	42.41%	0.47	-29.77**	-41.27**
SDW	0.58	0.26	0.42	41.42%	0.50	20.37**	-12.90**
RDW	0.52	0.11	0.31	44.03%	0.40	27.78**	-22.50**
MFVW	0.65	0.25	0.45	24.24%	0.46	2.93	-28.97**

** indicates significance at *P* < 0.01. The significance of MPH for each trait is based on one-sample *t*-test of offspring values and MPV. The significance of HPH for each trait is based on one-sample *t*-test of offspring values and HPV.

**Fig. 2:**
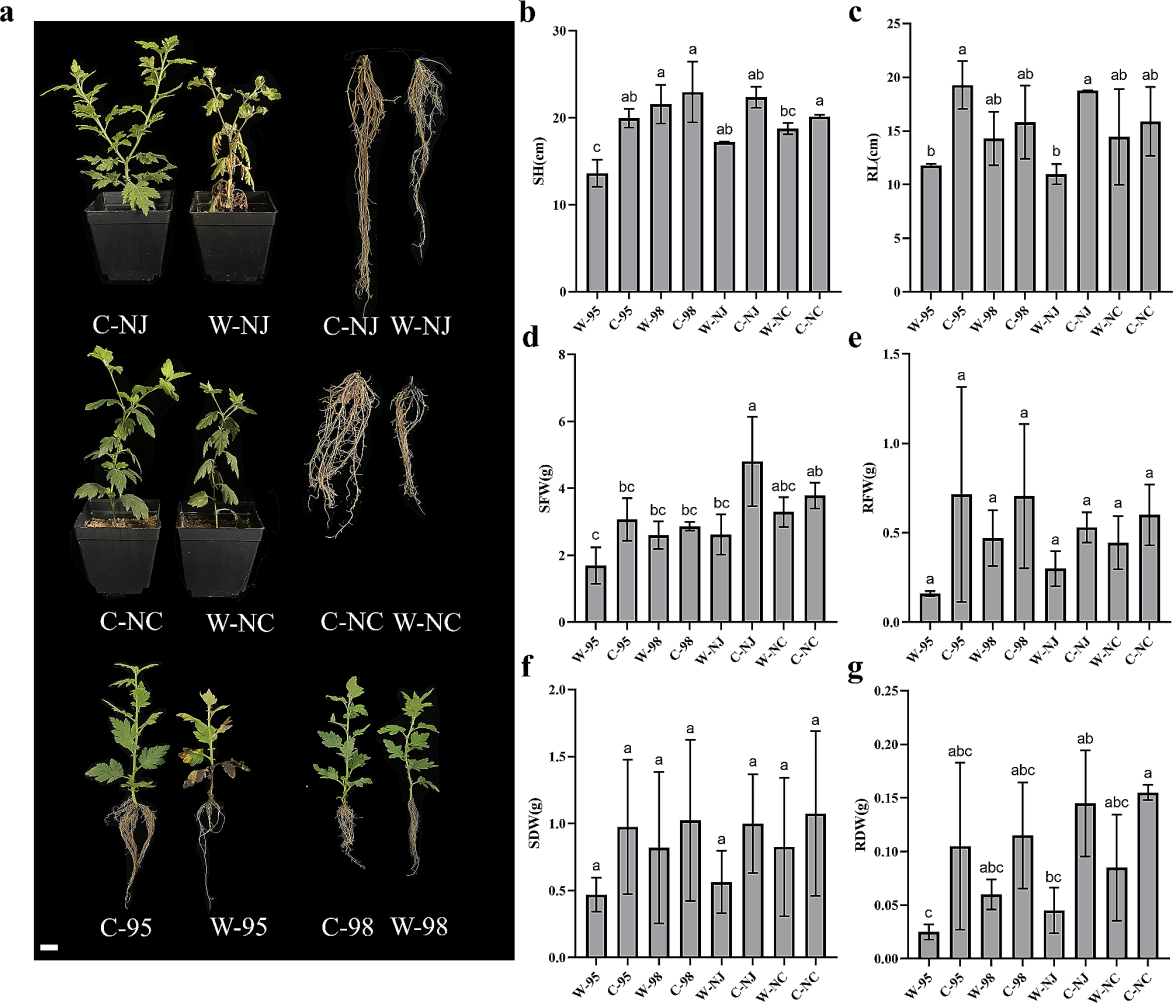
Phenotypic characteristics of F_1_ hybrids 95 and 98 and their parents. **a** plant phenotype of hybrids and parents after 9 days of waterlogging stress. Scale bar = 2 cm. **b-g** Statistical analysis of shoot height (SH), root length (RL), shoot fresh weight (SFW), root fresh weight (RFW), shoot dry weight (SDW), and root dry weight (RDW) of F_1_ hybrid 95 and 98 and their parents. Different letters indicate significant difference at *P* < 0.05 between individuals. Data represent mean values ± *SE*. The control group is prefixed with C; The 9-day waterlogging stress treatment group is prefixed with W; NC indicates female parent *C. indicum* (Nanchang), and NJ indicates male parent *C. indicum* (Nanjing)

Notably, line 95 had obvious yellowing, wilting, and even rotting of the lower leaves, and its root mass was significantly reduced compared to the control group. By contrast, line 98 showed no significant morphological changes (Fig. 2a). Heterosis analysis revealed that line 98 exhibited positive mid-parent heterosis for all waterlogging tolerance traits (Table S4, Fig. 2b-g), with Score showing the maximal value (58.73%), followed by RDW (32.56%). Additionally, line 98 displayed positive high-parent heterosis for all traits except RFW and RL, and the maximal high-parent heterosis rate was observed in Score (5.26%), indicating that line 98 behaved stronger waterlogging tolerance compared to the waterlogging-tolerant parent *C. indicum* (Nanchang). In contrast, line 95 had the lowest MFVW in this F_1_ population and exhibited negative mid-parent heterosis and high-parent heterosis for all traits (Table S4, Fig. 2b-g), indicating its weaker waterlogging tolerance than the waterlogging-sensitive parent *C. indicum* (Nanjing). Therefore, lines 98 and 95 were selected to study waterlogging tolerance heterosis further.

### RNA sequencing and quantitative real-time PCR validation

To study the differences in the response mechanisms to waterlogging stress between hybrid lines and parents, RNA of root tissues was extracted to construct 24 libraries. As a result, 47.94–54.93 million raw reads were yielded for each sample. After filtering the raw read data, we obtained 46.7–54.0 million clean reads, corresponding to 6.97–8.06 Gb of clean bases. The Q30 levels ranged from 91.87 to 93.05%, and the GC content ranged from 41.69 to 44.63%. A total of 119,965 unigenes were assembled, with lengths ranging from 201 to 1,446 bp and an average length of 808 bp. The N50 value was greater than 1,264 bp. Based on the annotation results from the Nr, KEGG, KOG, and SwissProt databases, a total of 61,549 unigenes were annotated in at least one database. The average Pearson’s correlation coefficient among the three samples was 0.9675 (Fig. S2), indicating that the transcript abundances of the biologically replicated samples were highly correlated. These results suggest the high quality of RNA-seq. Detailed information about the RNA-Seq data was provided in Table S5.

The reliability of the RNA-seq data was further validated using the qRT-PCR method. Six genes were randomly selected for validation, including AP2-ERF transcription factor (Unigene21365), late embryogenesis abundant (LEA) hydroxyproline-rich glycoprotein family (Unigene101624), DCD (Development and Cell Death) domain protein (Unigene43272), lipoxygenase 1 (Unigene3146), peroxidase superfamily protein (Unigene22039) and abscisic acid receptor PYL4-like (Unigene77240). As a result, all of the six genes examined showed similar expression profiles to the RNA-seq data (Fig. S3), confirming the reliability of the RNA-seq data.

### Identification of DEGs under waterlogging stress

**Fig. 3:**
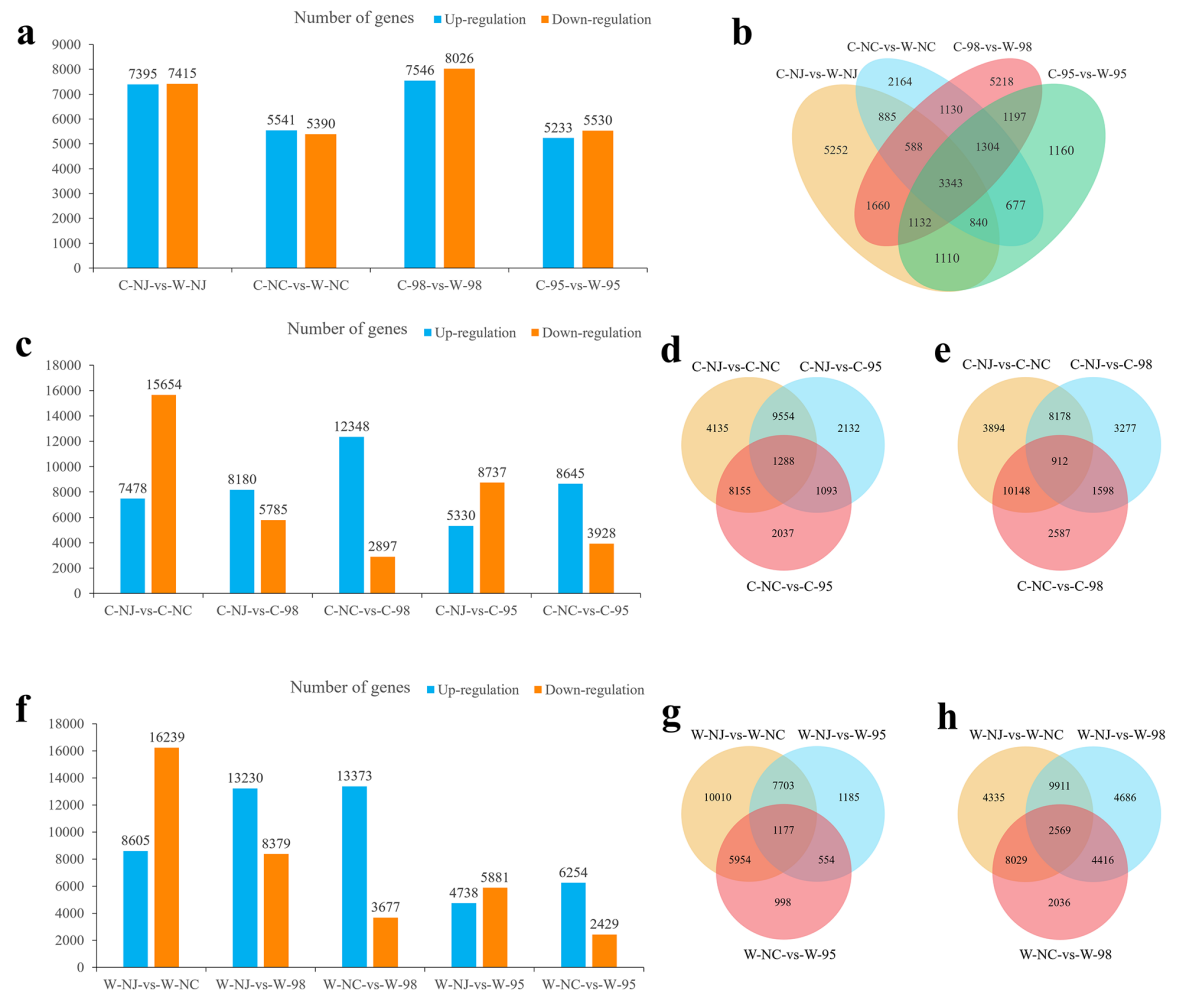
Basic analysis of differentially expressed genes. **a, b** Histogram and venn diagrams showing the number of up-regulated and down-regulated differentially expressed genes among hybrids 95 and 98 and their parents under waterlogging stress and control conditions. **c-e** Histogram and venn diagrams of up-regulated and down-regulated differentially expressed genes in paired comparison between hybrids and parents under control condition. **f-h** Histogram and venn diagrams of up-regulated and down-regulated differentially expressed genes in paired comparison between hybrids and parents under waterlogging stress condition. The control group is prefixed with a capital letter C; The 9-day waterlogging stress treatment group is prefixed with a capital letter W; NC, *C. indicum* (Nanchang); NJ, *C. indicum* (Nanjing)

Sixteen pairwise comparisons (C-98 vs. W-98, C-95 vs. W-95, C-NC vs. W-NC, C-NJ vs. W-NJ/C-NC vs. C-98, C-NJ vs. C-98, C-NJ vs. C-NC/W-NC vs. W-98, W-NJ vs. W-98, W-NJ vs. W-NC/C-NC vs. C-95, C-NJ vs. C-95, C-NJ vs. C-NC/W-NC vs. W-95, W-NJ vs. W-95, W-NJ vs. W-NC) were made to examine the DEGs (fold change ≥ 2 and FDR ≤ 0.05). In the comparisons between the control and waterlogging stress groups, a total of 15,572 (7,546 up-regulated and 8,026 down-regulated), 10,763 (5,233 up-regulated and 5,530 down-regulated), 10,931 (5,541 up-regulated and 5,390 down-regulated), and 14,810 (7,395 up-regulated and 7,415 down-regulated) DEGs were identified for line 98, line 95, NC, and NJ, respectively. Compared to the parental and highly waterlogging-sensitive line 95, the highly waterlogging-tolerant hybrid line 98 exhibited the maximal number of DEGs, indicating its strong response to waterlogging stress (Fig. 3a, b).In the pairwise comparisons of C-NC vs. C-98, C-NJ vs. C-98, and C-NJ vs. C-NC, 15,245 (12,348 up-regulated and 2,897 down-regulated), 13,965 (8,180 up-regulated and 5,785 down-regulated), and 23,132 (7,478 up-regulated and 15,654 down-regulated) DEGs were identified, respectively (Fig. 3c-e). In the pairwise comparisons of W-NC vs. W-98, W-NJ vs. W-98, and W-NJ vs. W-NC, 17,050 (13,373 up-regulated and 3,677 down-regulated), 21,582 (13,203 up-regulated and 8,379 down-regulated), and 24,844 DEGs (8,605 up-regulated and 16,239 down-regulated) were identified, respectively (Fig. 3f-h). In addition, more parental DEGs were discovered in line 98 under waterlogging stress, indicating line 98 might have different response mechanisms to waterlogging stress from those of its parents, and the related DEGs might accordingly explain the waterlogging tolerance heterosis.

Correlations between the hybrid and its parents in gene expression were investigated with cluster analysis using Cluster 3.0 software. The transcriptome profiles of line 95 were similar to *C. indicum* (Nanjing) under waterlogging stress and control conditions. The transcriptome profiles of line 98 were identical to *C. indicum* (Nanjing) (male parent) under control conditions, but it was more similar to *C. indicum* (Nanchang) (female parent) under waterlogging stress conditions (Fig. S4).

### Functional enrichment analysis of the common DEGs under waterlogging stress

**Fig. 4:**
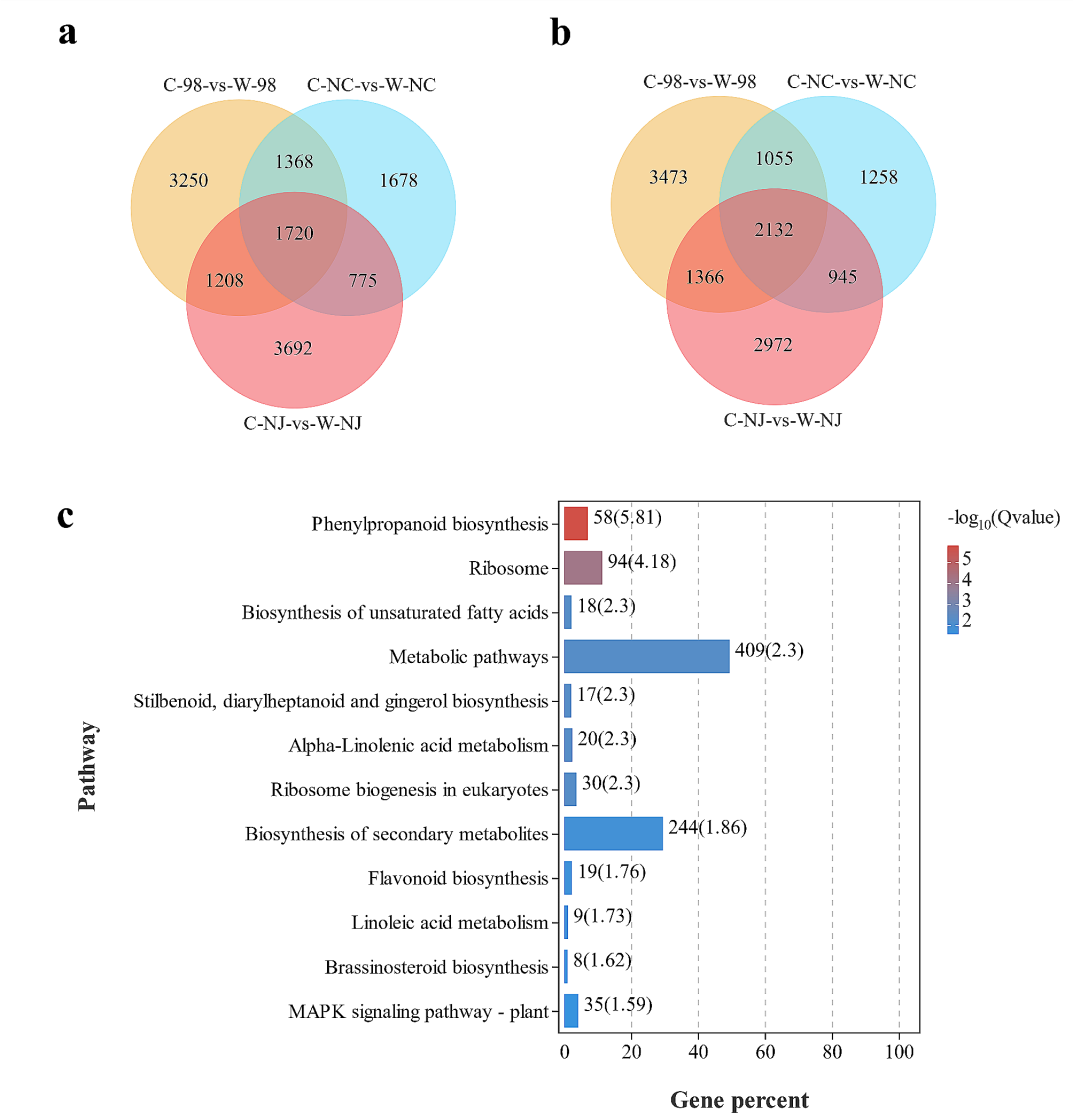
Basic analysis of differentially expressed genes and KEGG enrichment analysis of line 98 and its parents under waterlogging stress and control conditions. **a, b** Venn diagram of up-regulated and down-regulated differentially expressed genes. **c** KEGG enrichment map of 3,931 common DEGs of line 98. The numbers on the right side of the bar graph represent the number of genes contained in the corresponding KEGG pathway; The numbers in parentheses represent the significance level of the corresponding KEGG pathway (-Log_10_(Q value)). The KEGG enrichment map mentioned in the figure has been granted copyright permission by Kanehisa Laboratories

In the pairwise comparisons between the control group and the waterlogging stress group, 3,931 genes (1,720 up-regulated, 2,132 down-regulated) out of the total 15,572 DEGs were found to be in common among line 98 and the parents (Fig. 4a, b). The functional enrichment analysis uncovered that these common genes were involved in 55 biological processes (FDR ≤ 0.05), including several terms related to response to stimulus (GO:0050896), hormone (GO:0009725), stress (GO:0006950), etc. (Table S6). Further analysis of the common up-regulated genes showed that 63 GO biological processes were significantly enriched (Table S7), including various processes involved in plant responses to abiotic stress, particularly responses to stimulus (GO:0050896) and stress (GO:0006950), with 451 and 295 genes significantly enriched, respectively. Additionally, terms related to ethylene metabolism and hormone response, such as ethylene metabolic process (GO:0009692), cellular alkene metabolic process (GO:0043449), and response to hormone (GO:0009725), were also enriched. As for the common down-regulated genes, 45 GO biological process terms were significantly enriched, including processes related to RNA modification (GO:0009451), ribonucleoprotein complex biogenesis (GO:0022613), and organic substance transport (GO:0071702) (Table S8). The functional enrichment analysis of the common DEGs between line 98 and the waterlogging-tolerant parent *C. indicum* (Nanchang) revealed 14 significant biological process terms, such as phytoalexin biosynthetic process (GO:0052315), indole phytoalexin metabolic process (GO:0046217), and phytoalexin metabolic process (GO:0052314). However, no significant biological process terms were enriched in the specific DEGs of line 98 or its common DEGs with the waterlogging-sensitive parent *C. indicum* (Nanjing).

In the meantime, KEGG pathway enrichment analysis was conducted against the 3,931 common DEGs. The results revealed that 12 KEGG pathways involving a total of 827 DEGs, including phenylpropanoid biosynthesis, ribosome, biosynthesis of unsaturated fatty acids, metabolic pathways, stilbenoid, diarylheptanoid, and gingerol biosynthesis, α-linolenic acid metabolism, ribosome biogenesis in eukaryotes, and biosynthesis of secondary metabolites, etc., were significant enriched (Fig. 4c).

### Expression pattern analysis of DEGs between hybrids and parents

**Fig. 5:**
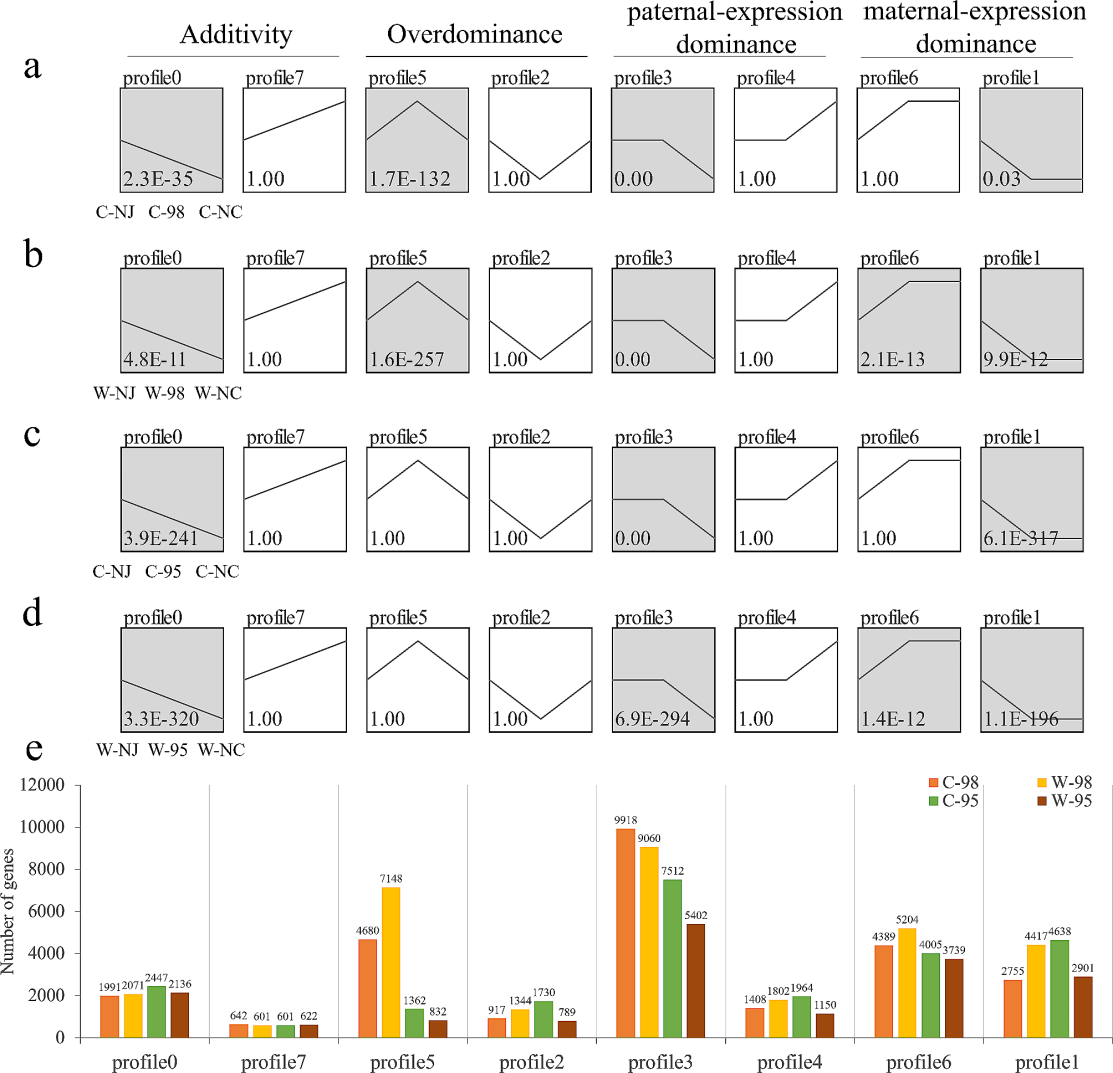
Analysis of gene expression trends in hybrids compared to parents under control and waterlogging stress conditions. **a, b** The gene expression genetic patterns of line 98. **c, d** The gene expression genetic patterns of line 95. The corresponding *P*-value is marked in the expression pattern diagram, and the significant expression patterns (*P* < 0.05) are marked in gray. **e** Statistical analysis of the number of genes contained in each expression pattern. The control group is prefixed with a capital letter C; The 9days waterlogging stress treatment group is prefixed with a capital letter W; the numbers after C and W represent biological repeated numbers; NC, *C. indicum* (Nanchang); NJ, *C. indicum* (Nanjing)

Based on the expression profiles of parents and hybrids, gene expression levels were categorized into eight groups, i.e., Profile 0 - Profile 7 (Fig. 5a-d). Profile 0 and Profile 7 represented additive expression, Profile 1, Profile 3, Profile 4, and Profile 6 represented dominant expression, and Profile 2 and Profile 5 represented overdominant expression, respectively. Trend analysis was performed using the DEGs between hybrids and parents under control and waterlogging stress conditions. Intriguingly, four expression patterns, namely, additivity (Profile 0), transgressive up-regulation (Profile 5), paternal-expression dominance (Profile 3), and maternal-expression dominance (Profile 1), were significantly enriched for line 98 (Q value ≤ 0.05) under control and waterlogging stress conditions. Additionally, line 98 displayed an enrichment of maternal-expression dominance pattern (Profile 6) under waterlogging stress. These non-additive expression patterns contained a total of 25,829 genes in line 98 under waterlogging stress, accounting for 39.41% of the total DEGs (65,547), which was much higher than that in line 95 (14,813 DEGs; 21.63%). Under control condition, line 98 showed the maximal number of DEGs in Profile 3 (9,918), followed by Profile 5 (4,680) (Fig. 5e). However, under waterlogging stress, the two maternal-expression dominance patterns (5,204 for Profile 6 and 4,417 for Profile 1) and overdominant expression pattern (7,148 for Profile 5) in line 98 possessed higher number of DEGs (Fig. 5b, e). These results suggested that the three non-additive expression patterns may play an important role in waterlogging tolerance heterosis in chrysanthemums, especially the overdominant expression pattern with the highest increase in genes after experiencing waterlogging stress. Under both control and waterlogging stress conditions, the highest number of DEGs in maternal expression dominance patterns was observed in line 95 (Fig. 5c, e). However, after being subjected to waterlogging stress, the number of genes was decreased in line 95 for all expression patterns except profile 7 (Fig. 5d, e).

### Comparative and functional enrichment analyses of non-additive genes in hybrids

**Fig. 6:**
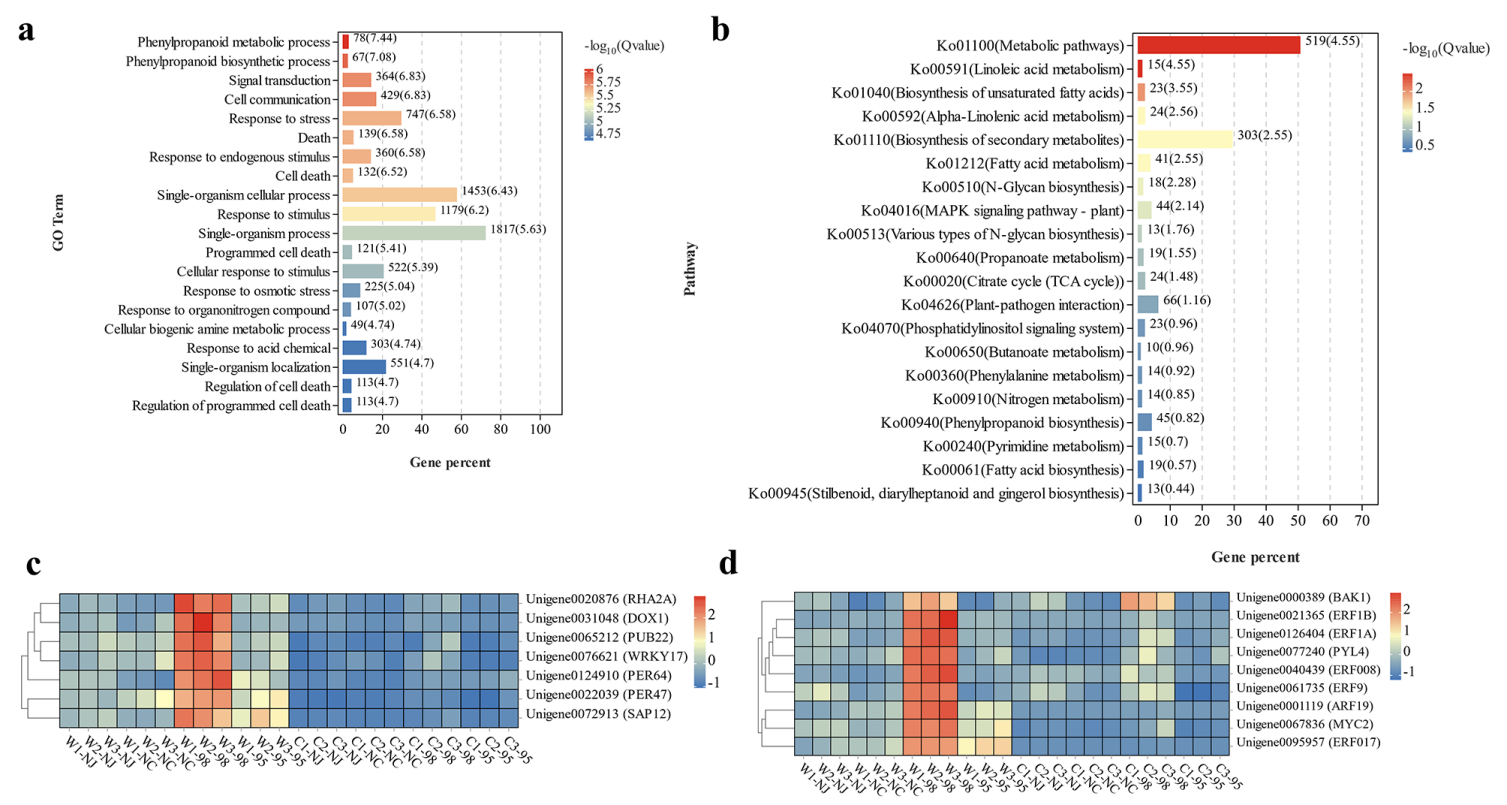
Enrichment analysis of 5,069 unique transgressive up–regulation genes and expression heat map of 16 candidate genes. **a** Enrichment of GO biological process terms for 5,069 unique transgressive up–regulation genes of line 98. Only the top 20 GO terms with the lowest Q value are shown. **b** KEGG enrichment of 5,069 unique transgressive up–regulation expression genes in line 98. Only the top 20 pathways with the lowest Q value are shown. The numbers on the right side of the bar graph represent the number of genes contained in the corresponding GO terms. The numbers in parentheses represent the significance level of the corresponding GO terms (-Log_10_(Q value)). The KEGG enrichment map mentioned in the figure has been granted copyright permission by Kanehisa Laboratories. **c** Expression heatmaps of 9 hormone response-related genes enriched in GO terms in response to the hormone. **d** Expression heatmaps of 7 plant stress resistance-related genes enriched in GO terms in response to stress. The legend at the right of the figure indicates the gene expression levels transformed by log_10_ (FPKM)

To further investigate the waterlogging tolerance heterosis in line 98, enrichment analyses of the DEGs showing transgressive upregulation (profile5) and the maternal expression dominance (profile6) under waterlogging stress were conducted. The results revealed that the DEGs in profile 5 were enriched in 189 GO biological process terms, of which some were related to waterlogging tolerance, including responses to osmotic stress (GO:0006970), response to stress (GO:0006950), stimulus (GO:0050896), and hormone (GO:0009725) (Table S9). However, the DEGs in Profiles 1 and 6 did not show significant enrichment in biological process terms. Under waterlogging stress condition, 5,069 out of the 7,148 transgressive up-regulation genes in line 98 were specific to waterlogging stress, which was assigned to 148 GO biological process terms with significant enrichment in waterlogging tolerance-related GO terms, including responses to stress (GO:0006950), stimulus (GO:0050896), osmotic stress (GO:0006970), hormone (GO:0009725), and external stimuli (GO:0009605). The top 20 significantly enriched GO terms and KEGG pathways are shown in Fig. 6a and b. The response to stress term contained 747 genes with an overlap of 55 genes within the 1,720 common up-regulated DEGs (Table S10). The response to the stimulus term contained 1,179 genes, with an overlap of line 95 genes within the common up-regulated DEGs. Furthermore, many stress-related genes were found among these overlapped genes, such as *PUB23-like* (Unigene0065212), *WRKY17* (Unigene0076621), *RHA2A* (Unigene0020876), and *SAP12* (Unigene0072913), as well as three genes involved in reactive oxygen species defense: *DOX1* (Unigene0031048), *PER64* (Unigene0124910), and *PER47* (Unigene0022039). The heatmap of expression levels demonstrated that the above genes exhibited higher expression levels in line 98 compared to that in line 95 and two parents, indicating their potential roles in the waterlogging tolerance heterosis (Fig. 6c). Notably, *SAP12* and *DOX1* showed a 12-fold and 10-fold up-regulation under waterlogging treatment, respectively. Among the 120 genes covered by the response to hormone (GO:0009725) term, five transcription factors related to ethylene signaling were identified, including *ERF008* (Unigene0040439), *ERF1A* (Unigene0126404), *ERF1B* (Unigene0021365), *ERF9* (Unigene0061735), and *ERF017* (Unigene0095957). Specifically, *ERF017* and *ERF1B* showed 38-fold and 7-fold up-regulation under waterlogging stress, respectively. In addition to ethylene, several enriched DEGs related to other hormones were also discovered, such as auxin-responsive factor *ARF19* (Unigene0001119), jasmonic acid signaling-related transcription factor *MYC2* (Unigene0067836), abscisic acid receptor *PYL4-like* (Unigene0077240), and brassinosteroid-associated gene *BAK1* (Unigene0000389). These hormone-responsive genes also exhibited significantly higher expression levels in line 98 (Fig. 6d), indicating their potential contribution to waterlogging tolerance. Overall, through RNA-seq, we provided functional links of heterosis to waterlogging tolerance in *C. indicum*. Nevertheless, these potential genes still need more evidence to support them in further detail.

## Discussion

The utilization of heterosis has long been one of the main objectives of chrysanthemum breeders. Although heterosis has been widely exploited in improving the tolerance to biotic and abiotic stresses of cultivated chrysanthemum via intraspecific and interspecific crosses [40, 56], the molecular and genetic mechanisms underlying the phenomenon remain largely unknown, which might be attributed to the complicated genetic background and high heterozygosity of cultivated chrysanthemum. *C. indicum* is an ideal model species of hexaploidy chrysanthemum owing to its high phenotypic diversity and close relationship with chrysanthemum [35]. Liu et al. (2012) proposed that *C. indicum* (Nanjing) played an important role in the origin of cultivated chrysanthemum according to the phylogenetic analysis of single-copy nuclear gene and chloroplast DNA sequences [57]. A recent study also revealed that *C. indicum* (Nanjing) is more closely related to cultivated chrysanthemum based on whole genome resequencing [36]. In addition, heterosis is strongly correlated with the genetic distance between parents in studies on rice [58], broccoli [59], and *Arabidopsis* [60]. Therefore, *C. indicum* (Nanjing) and *C. indicum* (Nanchang), which exhibited significant differences in waterlogging tolerance, were selected in the present study as parents to generate an F_1_ population for heterosis analysis of waterlogging tolerance. The results showed that all the measured waterlogging tolerance traits were widely segregated within the F_1_ population, with the coefficients of variation for root-related traits significantly higher than those for shoot-related traits (Table S2). This indicates that different genotypes exhibit more considerable differences in roots than shoots after being subjected to waterlogging stress. It also highlights the importance of root system growth in the phenotypic evaluation of waterlogging tolerance, consistent with previous findings [38].

Using transcriptome sequencing to analyze DEGs between hybrids and parents is an effective way to explore possible molecular mechanisms for heterosis. In the pairwise comparisons between *C. indicum* (Nanjing), *C. indicum* (Nanchang), and line 98, it was found that the maximum number of DEGs was detected between the two parents under both control and waterlogging stress (23,132 and 24,844). There were fewer DEGs between line 98 and *C. indicum* (Nanjing) (13,965) under control conditions, while fewer DEGs between line 98 and *C. indicum* (Nanchang) (17,050) (Fig. 3c, f) were observed under waterlogging stress. This result was consistent with the clustering analysis of all transcriptions, in which line 98 was closely clustered with *C. indicum* (Nanjing) under control conditions, whereas it was separated under stress (Fig. S4). This phenomenon of genes in hybrids tending to be biased towards specific parents in their expression has also been reported in maize, *Arabidopsis*, and rice [61−63]. The interaction between waterlogging-responsive genes inherited from different parents in line 98 might activate or suppress more responsible genes. Additionally, stress-tolerance genes inherited from *C. indicum* (Nanchang) may exhibit distinct expression patterns in hybrids. These issues might contribute to the waterlogging tolerance heterosis observed in line 98.

Reasonable gene selection and discovery are critical aspects of transcriptome analysis. To identify waterlogging-tolerant-related DEGs, three pairwise comparisons (C-98 vs. W-98, C-NC vs. W-NC, C-NJ vs. W-NJ) were performed. A total of 3,931 common DEGs and line 98 specific genes were identified in this study (Fig. 3a, b). GO enrichment analysis demonstrated that these parental common genes were mainly involved in several processes directly related to plant stress responses (Table S6). However, the DEGs specific to line 98 did not enrich any biological process terms. This suggests that the common DEGs play an important role in the waterlogging tolerance heterosis of line 98, consistent with previous studies [29, 31, 32, 64] that focused on shared genes for heterosis analysis. Further enrichment analysis revealed that among the up-regulated shared genes, 295 genes were enriched in the “response to stress” (GO:0006950) term, and 451 genes were enriched in the “response to stimulus” (GO:0050896) term, which might play a crucial role in response to waterlogging stress and contribute to the waterlogging tolerance heterosis observed in line 98. This further confirms that the expression levels of common genes related to waterlogging stress response in line 98 have been changed under the combined action of the parental genomes, thereby enhancing its waterlogging tolerance.

Non-additive effects have been proven to play a vital role in the formation of plant heterosis [65]. In this study, trend analysis was conducted on the DEGs of lines 98 and 95 under control and waterlogging stress conditions. Among the five significant expression patterns identified in line 98, the number of genes assigned to the transgressive up-regulation pattern increased dramatically under waterlogging stress, followed by two maternal-expression dominant patterns (Fig. 5e). Further GO enrichment analysis revealed that the genes belonging to the transgressive up-regulation pattern were significantly enriched in multiple stress response-related biological process terms, indicating the important role of transgressive up-regulation genes in the waterlogging tolerance heterosis of chrysanthemum. Similar findings have been reported in previous studies on rice [66] and maize [67], where multiple key yield-related heterosis loci exhibited transgressive up-regulation. Studies on biomass heterosis in cotton [68] and tobacco [69] also found that the primary expression pattern of DEGs in hybrids was transgressive up-regulation. The transcriptome analysis of maize hybrid An’nong 591 also discovered that the number of overdominance genes increased more than 2.5 times under heat stress [32], which is consistent with the results of this study. GO enrichment analysis was conducted on the 5,069 waterlogging stress-specific transgressive up-regulation genes and compared them with the up-regulated parental common genes previously screened out. This further led to the identification of transgressive up-regulation parental common genes that are responsible for waterlogging stress response. The results showed that 148 GO biological process terms were significantly enriched, including responses to organic stress (GO: 0006970), endogenous stimuli (GO: 0009719), chemical (GO: 0042221), stress (GO: 0006950), and stimuli (GO: 050896). Among them, 55 genes were assigned to responses to stress terms, and 95 were assigned to responses to stimulus terms (Table S10). Furthermore, seven out of the 55 genes were selected. *PUB23-like* (Unigene0065212) [70] and *WRKY17* (Unigene0076621) [71] have been shown to play a role in plant stress tolerance studies. *RHA2A* (Unigene0020876) has also been confirmed to be associated with drought and salt tolerances in previous studies on soybeans [72] and rice [73]. The expression level of the oxidative stress-related gene *SAP12* (Unigene0072913) [74, 75] in super line 98 increased 12-fold after waterlogging stress treatment. *DOX1* (Unigene0031048), *PER64* (Unigene0124910), and *PER47* (Unigene0022039) are associated with plant reactive oxygen species. The strong waterlogging tolerance of line 98 may result from the combined action of different types of stress response pathways. Ethylene plays an important role in plant response to waterlogging stress, and previous transcriptome analyses have identified ethylene-responsive genes that are important for chrysanthemum waterlogging tolerance [43]. Five transcription factors related to ethylene signaling were recognized in the waterlogging stress-specific transgressive up-regulation genes. Among them, *ERF1B* (Unigene0021365) and *ERF017* (Unigene0095957) showed the most robust responses to waterlogging, with 7-fold and 38-fold up-regulation, respectively. These genes may also participate in the formation of waterlogging tolerance heterosis and are worth further investigating.

## Conclusion

In this study, RNA-seq was used to elucidate the differences in the global gene expression profiles of *C. indicum* (Nanjing), *C. indicum* (Nanchang), as well as their positive over-dominant hybrid line 98 and negative over-dominant hybrid line 95 under control and waterlogging stress conditions. Trend analysis was also conducted to depict their gene expression patterns and explore the mechanisms underlying the waterlogging tolerance heterosis in chrysanthemums. The study ultimately highlighted the significant roles of transgressive up-regulation expressed genes and parental common up-regulated genes in the waterlogging tolerance heterosis of the hybrids. These findings provide new insights into the mechanism of heterosis and contribute to future heterosis breeding and molecular improvement of waterlogging tolerance of chrysanthemum.

### Electronic supplementary material

Below is the link to the electronic supplementary material.

Supplementary Material 1

Supplementary Material 2

Supplementary Material 3

Supplementary Material 4

Supplementary Material 5

Supplementary Material 6

Supplementary Material 7

Supplementary Material 8

Supplementary Material 9

Supplementary Material 10

Supplementary Material 11

Supplementary Material 12

Supplementary Material 13

Supplementary Material 14

Supplementary Material 15

## Data Availability

RNA-seq data of this study can be found at the National Center for Biotechnology Information (https://www.ncbi.nlm.nih.gov/bioproject/) with the BioProject ID PRJNA1013215.

## Abbreviations

WI: Waterlogging tolerance index
MFVW: Membership function value of waterlogging tolerance traits
MPV: Mid-parent value
HPV: High-parent value
RPKM: Reads per kilobase of transcript per million mapped reads
GO: Gene ontology
KEGG: Kyoto encyclopedia of genes and genomes
QTL: Quantitative trait locus
NR: Non-redundant protein sequence database
KOG: Clusters of orthologous groups for eukaryotic complete genomes
FDR: False discovery rate
