# Supplementary figures and images for: Comparative transcriptome analysis provides molecular insights into heterosis of waterlogging tolerance in Chrysanthemum indicum
Source: BMC Plant Biol. 2024 Apr 10;24:259. doi: 10.1186/s12870-024-04954-4 (PMC11005212; doi:10.1186/s12870-024-04954-4)

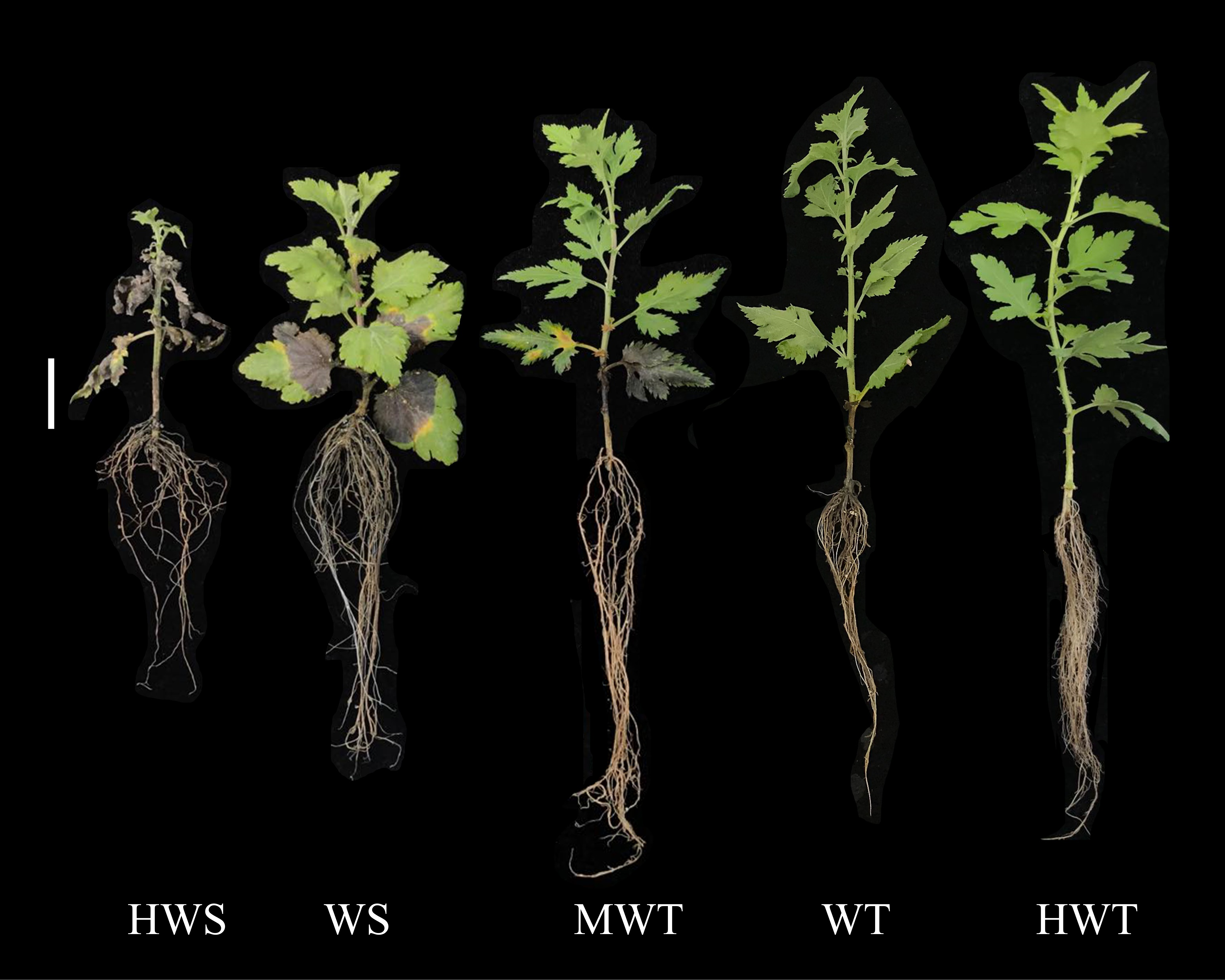

Supplement: Supplementary file 4 — Supplementary Material 4 [file 12870_2024_4954_MOESM4_ESM.png]

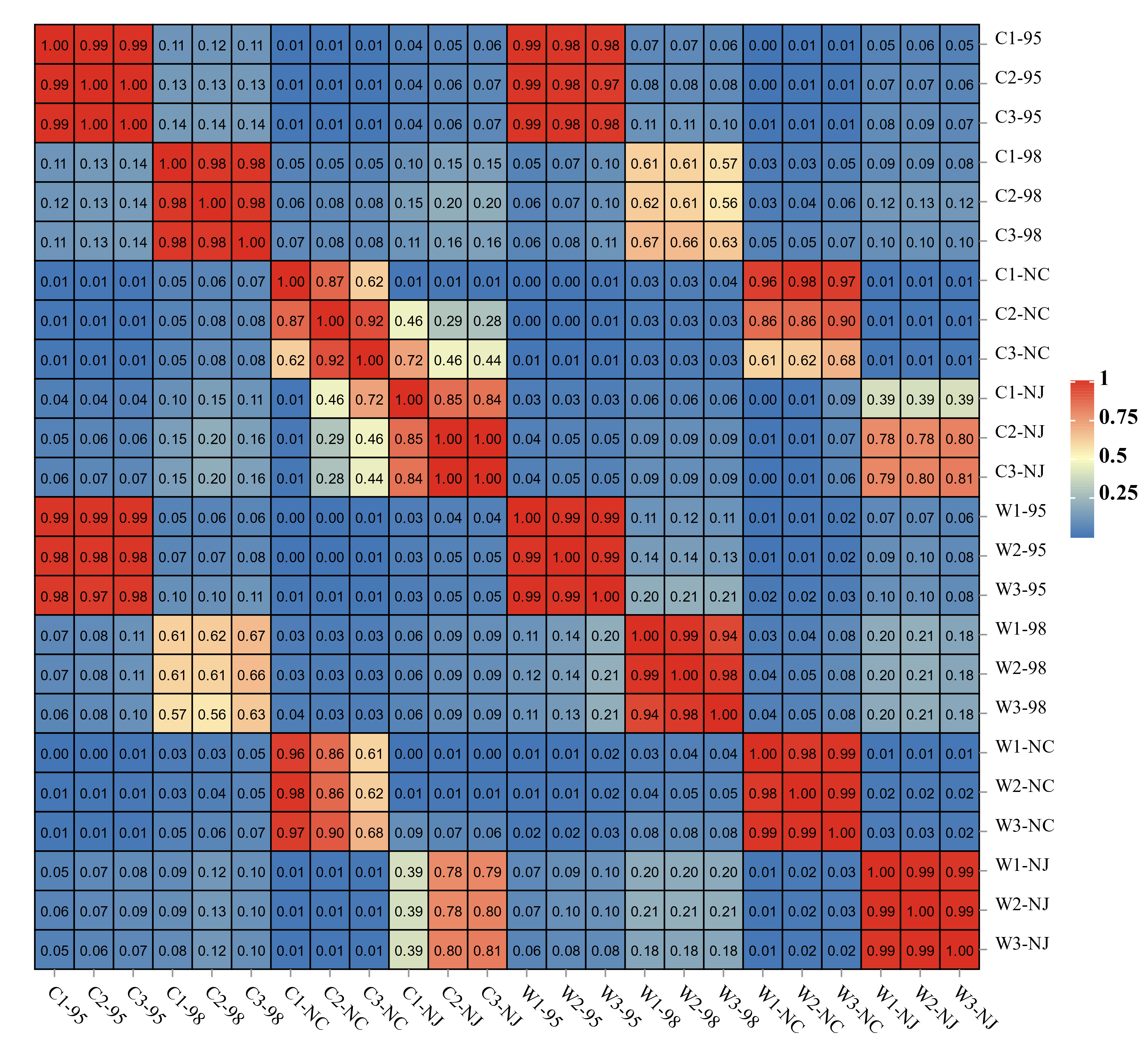

Supplement: Supplementary file 5 — Supplementary Material 5 [file 12870_2024_4954_MOESM5_ESM.png]

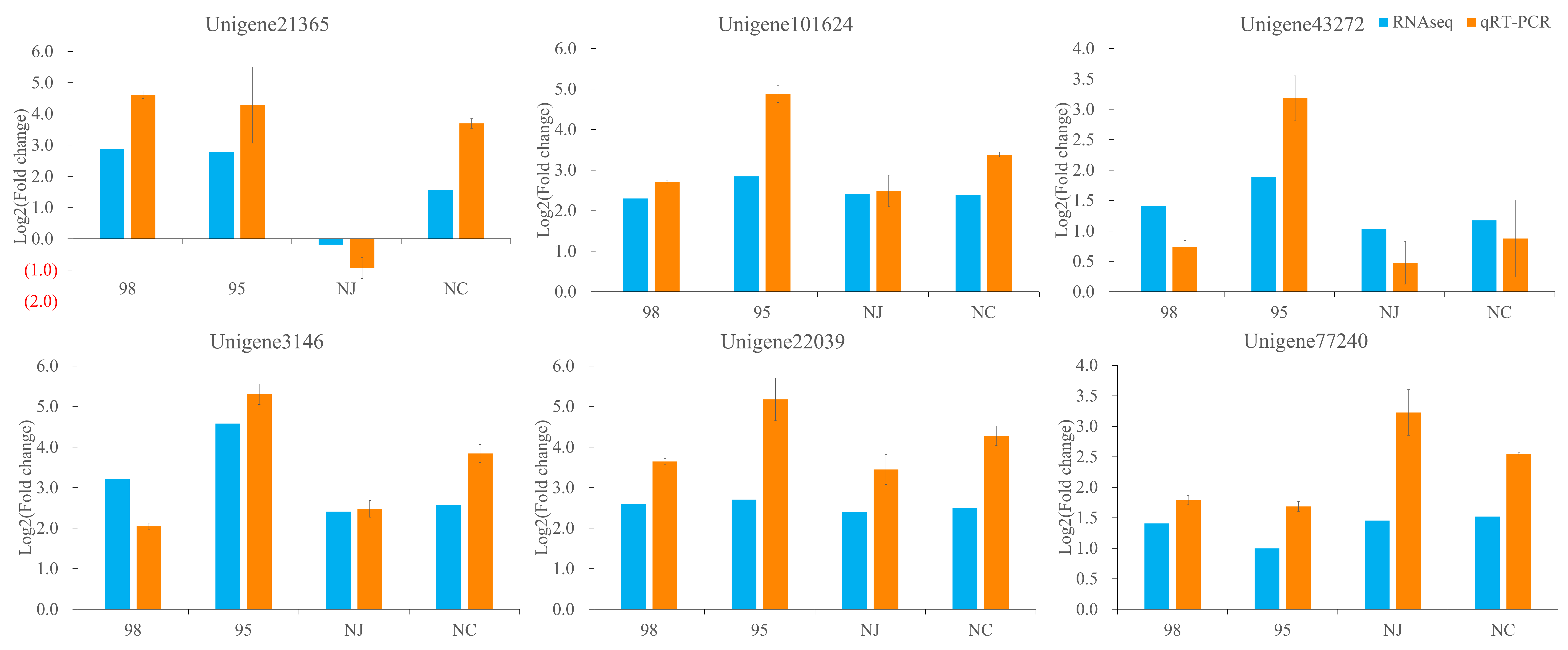

Supplement: Supplementary file 6 — Supplementary Material 6 [file 12870_2024_4954_MOESM6_ESM.png]

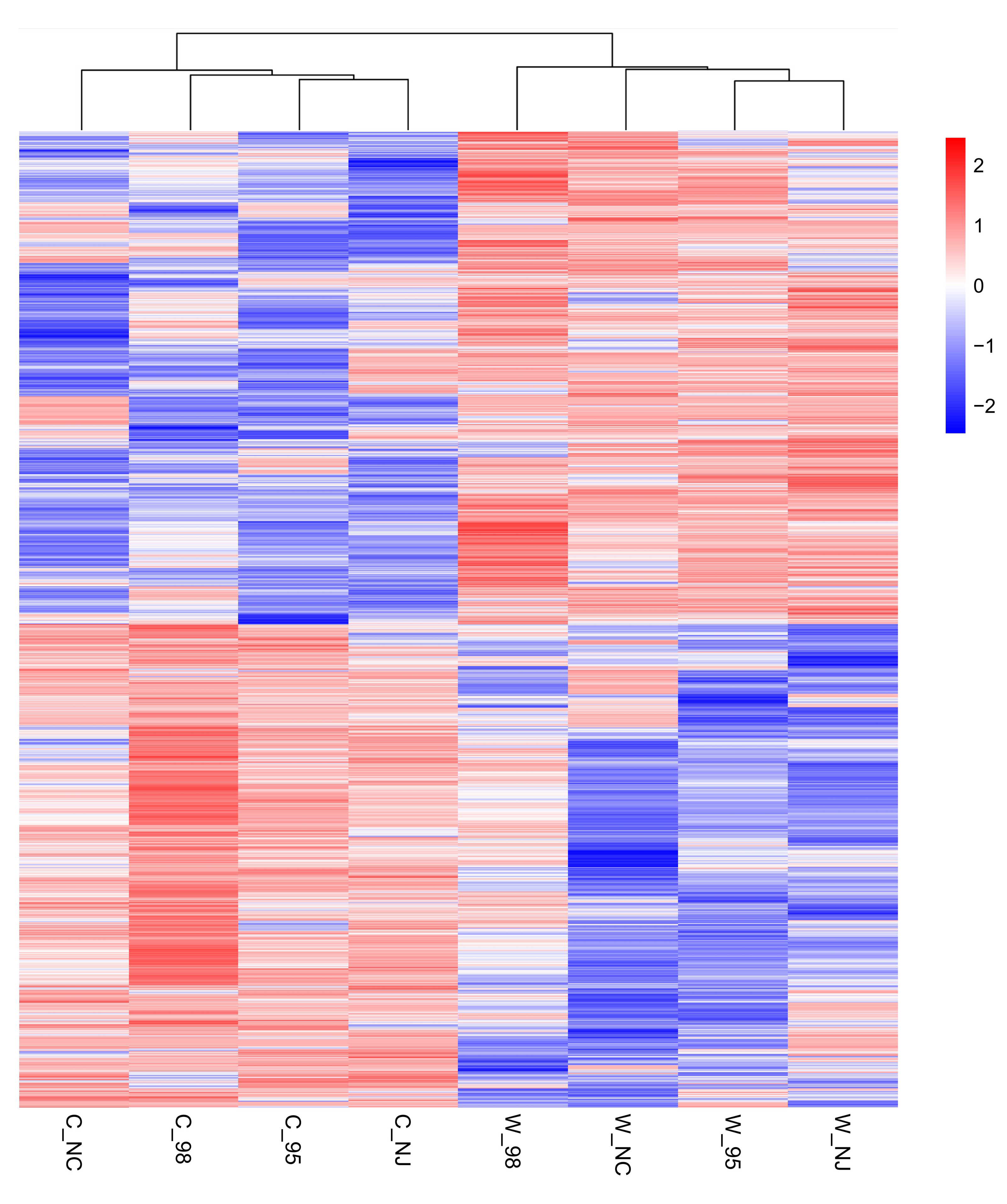

Supplement: Supplementary file 13 — Supplementary Material 13 [file 12870_2024_4954_MOESM13_ESM.png]
