# Supplementary material for: Comparative transcriptome analysis provides molecular insights into heterosis of waterlogging tolerance in Chrysanthemum indicum
Source: BMC Plant Biol. 2024 Apr 10;24:259. doi: 10.1186/s12870-024-04954-4 (PMC11005212; doi:10.1186/s12870-024-04954-4)
Supplement: Supplementary file 14 — Supplementary Material 14 [file 12870_2024_4954_MOESM14_ESM.docx]

**Fig. S1** The performance of five representative F_1_ offspring after 9 days of waterlogging stress treatment. The scale is 2 cm.

**Fig. S2** Correlation heat map of sample relationships between parents and hybrids 98 and 95. The control group is prefixed with a capital letter C; The 9-day waterlogging stress treatment group is prefixed with a capital letter W; the numbers after C and W represent biological repeated numbers; NC, *C. indicum* (Nanchang); NJ, *C. indicum* (Nanjing).

**Fig. S3** Quantitative real-time PCR (qRT-PCR) validation and RNA-seq data of 6 selected DEGs in hybrids and parents. The *y*-axis indicates the log_2_-transformed fold change of each DEG under the denoted conditions relative to control. Values of qRT-PCR validation are presented as the Log_2_(Fold Change) ± SE.

**Fig. S4** Heat map and hierarchical clustering analysis of all transcriptions of hybrids and parents under control and waterlogging stress conditions. The color key represents FPKM normalized log_10_ transformed counts. The control group is prefixed with a capital letter C; The 9-day waterlogging stress treatment group is prefixed with a capital letter W; NC, *C. indicum* (Nanchang); NJ, *C. indicum* (Nanjing).
